# Reflections on Identity and Role Navigation of Continuous Medical Education Tutors in General Practice

**DOI:** 10.1111/tct.70357

**Published:** 2026-02-06

**Authors:** Ivana Keenan, Aileen Barrett, Illona Duffy, Laoise Byrne, Claire McNicholas, Gillian Doran, Stephanie Dowling

**Affiliations:** ^1^ Irish College of GPs Dublin Ireland; ^2^ University College Dublin Dublin Ireland

**Keywords:** CME tutors, general practice, professional identity, role strain, small group learning

## Abstract

**Background:**

General practitioners (GPs) who take on the role of continuous medical education (CME) tutors play a vital part in facilitating small group learning among their peers. Despite the importance of this role, the personal experiences, motivations and unique challenges of CME tutors remain largely unknown. This study aimed to provide further insight into CME tutors' reflections on their role and professional identity formation.

**Methods:**

A qualitative research approach, supported by two theoretical frameworks—identity and role theory—was employed. Thirteen CME tutors across Ireland participated in semi‐structured interviews conducted via Zoom. The interviews were analysed thematically.

**Findings:**

Participants primarily identified themselves as GPs, but enthusiasm for peer learning and education led them to become CME tutors. They viewed their CME tutor role as deeply rewarding and enriching their clinical practice, reflective skills and identity as both doctors and educators. As CME tutors, they also embraced the role of *‘*go‐to’ person: being perceived as someone reliable and consistently supportive. The overlap between internal and external expectations often led to role strain, which was intensified by the informal expansion of role into areas beyond their remit, including helping with personal or practical issues of peers. Yet, our participants felt that the role of CME tutor embraced their personal and professional development, strengthening their commitment to medicine and education.

**Conclusion:**

The career of CME tutors in Ireland was highly valued among participants; yet, additional support is needed to address role‐related challenges and ensure the long‐term attractiveness and sustainability of this vital professional path.

## 1 Introduction

Continuing medical education (CME) is defined as ‘any activity that serves to maintain, develop or increase the knowledge, skills and professional performance and relationships that a physician uses to provide services for patients, the public or the profession’ [1]. Hence, CME is often viewed as a lifelong learning process necessary for the professional development of medical providers [1, 2, 3].

In general practice, CME aims to enhance the quality of healthcare provided to patients while supporting general practitioners (GPs) in improving their medical knowledge, skills and clinical practice [4, 5, 6]. The positive impacts of CME on GPs have also been noted, including reduced burnout, improved job satisfaction, increased motivation and a strengthened sense of competence [7, 8, 9, 10]. Previous research has highlighted that CME tutors play a central role in supporting CME. The role of CME tutors revolves around guiding the translation of knowledge into practice while designing and facilitating interactive learning sessions [8, 11, 12]. To successfully fulfil the role, CME tutors often possess diverse skills to foster a safe learning space and an inclusive peer environment while enhancing professional growth [8, 11, 12].

In Ireland, CME small group learning (SGL) was initiated in the early 1980s; however, the network expanded to help GPs meet their statutory obligation to participate in a Professional Competence (PCS) Scheme [13]. Organised in local areas, CME‐SGL groups represent a core support for GPs, primarily facilitating the delivery of continuing education to inform best practice and effective patient care while fulfilling Medical Council requirements [13]. Currently, CME‐SGL is delivered by 43 CME tutors, recruited nationally through the Irish College of GPs and based locally throughout the country. CME tutors coordinate peer‐group interactive learning, where each tutor is responsible for an average of four groups, each comprising 12 frequent attenders who gather approximately seven to eight times annually. The role expectations of CME tutors in Ireland revolve around the educational and organisational aspects of the CME‐SGL groups, including coordinating the groups' meetings, delivering educational sessions that support clinical and personal development and evaluating the benefits of the CME‐SGL programme to individual participants. Funding for this programme is provided by key health organisations, including the Health Service Executive (HSE), the National Doctors Training and Planning (NDTP) Programme and the Irish College of GPs.

In recent years, an unprecedented demand for new members to join CME‐SGL groups has led to significant pressure to expand the CME‐SGL network. This expansion has introduced two key challenges: first, a shortage of available CME tutors, with recruitment efforts falling short of meeting the rising demand; and second, existing tutors, while often willing to take on additional groups, have faced growing workload pressures and role expectations that exceed their formal contractual obligations.

Therefore, the focus of this study was to understand the working experiences of CME tutors, including their reflections on the role, pressures and responsibilities. Our specific research questions were as follows:

1. What are CME tutors' perspectives and reflections on their professional identity?
2. How do CME tutors prioritise their roles and balance expectations and potential role tensions?

## 2 Methods

### 2.1 Aim, Design and Setting of the Study

As qualitative researchers, we chose to approach this study from an interpretivist epistemological perspective, attempting to capture and represent diverse individual narratives while also reflecting on our own roles in supporting current and future CME tutors.

The dual theoretical approaches of professional identity [14, 15] and role theory [16] were employed to frame this study. As CME tutors often navigate multiple, sometimes conflicting roles (including those of clinician, educator and mentor), the identity theory helped explore professional identities and balance amid potential tensions. Conversely, the role theoretical approach provided a valuable framework for understanding CME tutors' experiences during SGL by examining how they navigate beliefs, responsibilities and interactions within the group. The application of both theoretical concepts aimed to provide deeper insight into the complex role of CME tutors in a healthcare setting and to help us answer our research questions about what CME tutors' reflections reveal about their professional identity and how they prioritise and balance role expectations and tensions.

### 2.2 Study Participants and Recruitment

A purposive recruitment strategy was employed for this study, and only CME tutors with at least 1 year of tutoring experience were invited to participate, ensuring that the potential participants were sufficiently familiar with the role. Additionally, CME tutors located in both urban and rural areas were recruited to ensure that the diversity of working experiences and perceptions of the role was captured.

The invitations to participate in the study were facilitated through the CME National Directors (ID, LB and CMcN) and the Irish College of GPs (GD), who distributed the study information flyers to CME tutors who met the inclusion criteria. The information flyer contained an information form and the email addresses of the primary researchers (IK and AB). The information form provided comprehensive information on the study's purpose, procedures and potential risks and benefits.

### 2.3 Data Collection

Semi‐structured interviews were used as a data collection tool, allowing the researcher to delve into participants' personal narratives and reveal the social context and meaning they attach to their experiences [17]. The interview topic guide is available in Appendix A.

The interview questions were initially piloted with a senior CME tutor (ID) who provided valuable feedback, resulting in minor changes to the questions.

In total, 13 CME tutors participated in semi‐structured interviews; 10 were currently in the role, and three had recently retired. Prior to the interviews, each participant provided written and oral consent for the interview to be recorded and transcribed. All semi‐structured interviews were conducted online via the Zoom platform and lasted between 32 and 60 min. The primary researcher (IK) conducted and transcribed the interviews.

### 2.4 Data Analysis

Thematic analysis was employed to identify patterns and common themes within and across the datasets [18]. Firstly, two researchers (IK and AB) independently completed the initial coding of two interviews and developed a coding framework. Subsequently, IK coded all transcripts and applied inductive thematic analysis, following the steps proposed by Braun and Clark [18]. The process of coding and organising themes was carried out using NVivo 15 computer software.

### 2.5 Ethical Approval

Ethics approval for the study was obtained from the Irish College of GPs Research Ethics Committee (ICGP_REC_2024_ 2803). A strong emphasis was placed on voluntary participation and the participant's right to withdraw from the research at any point without prejudice or giving reasons for that decision. Before analysis, all transcripts were de‐identified to ensure the participants' anonymity and confidentiality.

## 3 Findings

Overall, 13 CME tutors participated in this study. Table 1 provides a more detailed breakdown of the participants' characteristics and work commitments.

**TABLE 1:** Profile of the participants.

	%	*N*
Gender		
Male	53.8	7
Female	46.2	6
Location		
Rural	23.0	3
Urban	38.5	5
Mixed^a^	38.5	5
Years working in CME		
3–7	38.5	5
8–11	23.0	3
11+	38.5	5
No. of CME groups		
3	53.8	7
4	15.4	2
5	7.7	1
6	23.1	3
Working hours a week		
5–7	61.5	8
7+	38.5	5

^a^ Mixed general practices term refers to practices located in an area that has both urban and rural characteristics.

**FIGURE 1:**
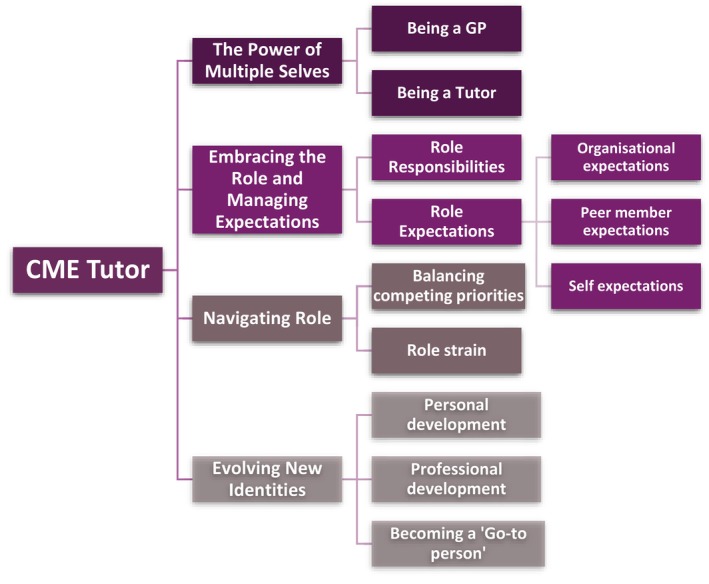
Thematic framework, including main themes and subthemes, derived from the thematic analysis of CME tutors' experiences of their role.

The thematic analysis revealed four main themes: ‘The power of multiple selves’, ‘Embracing the role and managing expectations’, ‘Navigating role’ and ‘Evolving new identities’ (Figure 1).

### 3.1 The Power of Multiple Selves

At the time of the study, most participants held various professional roles, including working as a GP, CME tutor, GP trainer or university professor and engaging in various special interest areas. However, ‘being a GP’ and ‘being a CME tutor’ were identified as their core identities, which influenced one another, creating a dynamic, evolving sense of self.

Essentially, participants' narratives revealed that *being a GP* was an inseparable part of their identity and had priority over other professional roles they held. Working as a GP represented their occupation and vocation equally as they saw themselves as ‘Always doctor, first … because that's who I am’ (CMET03) and ‘I just loved every minute of working as a GP’ (CMET12).

Yet, their interest in education and desire to collaborate with their peers led to a strong dedication to the CME tutor role. Although working as a CME tutor requires managing various duties that demand significant time and travel, the participants reiterated that one undertakes the role not merely out of interest and financial benefits, but out of passion and an overall sense of accomplishment:

It's my passion, too; if you don't have passion for it, you can't do it. It's quite hard work, the salary is not that great, the time is unsocial, so you need to love it. That's why tutors are good at their jobs: because if they are tutors, they love it. (CMET01)

Most CME tutors disclosed that tutoring drove their continuous learning, including learning new materials and gaining knowledge from peer engagements and discussions, stimulating their professional growth, and clinical skills, as they felt that ‘you're constantly upskilling your clinical skills and your knowledge. And you're constantly challenging your attitudinal stuff, and I think you're growing instead of revolving’ (CMET10).

CME tutors expressed that the benefits of this role included not only staying informed on emerging primary care guidelines and practices but also fostering meaningful interactions with their peer members. Exposure to peer dynamics, collaborative learning and sharing diverse experiences further enhanced their listening and communication skills, essentially making them feel as if they were becoming ‘a better GP’:

I learn loads from other GPs, how they do things, how they engage with patients, how they learn from their patients, how they put themselves into their patients' shoes …. (CMET05)

### 3.2 Embracing the Role and Managing Expectations

To better understand CME tutors' working experiences, the study further investigated how they prioritise their roles and expectations. The participants disclosed that the CME tutor role encompasses a comprehensive commitment to the educational and organisational aspects of the CME‐SGL groups, including a broad range of responsibilities: organising, preparing and facilitating the group meetings, providing a syllabus within the range of therapeutic and clinical topics, evaluating and planning meetings and programmes and attending CME workshops, among various administrative duties. Facilitating peer learning was seen as the primary responsibility, as all participants highlighted that they do not see themselves as teachers, rather facilitators, who promote peer learning, but also learn themselves: ‘I always tell them I'm here to learn as well, and I told them I'm learning from Gurus, you know, people who have loads of experience there’ (CMET01). Group learning was facilitated by incorporating everyone's voices, focusing on local challenges and solutions and linking key discussion points to current guidance. CME tutors believed that integrating all these aspects into the meetings would equip peer members with practical knowledge vital for patient care, as one tutor highlighted, ‘they apply the new knowledge in their practice, and that's the aim—better patient care’ (CMET01).

The role of CME tutor also brought overlapping layers of expectations, arising from CME directors, CME members and CME tutors themselves. The organisational expectations were briefly discussed, with a primary focus on encouraging and facilitating peer members' attendance, while maintaining high‐quality and engaging sessions that meet their educational and practical needs and overall CME standards.

Peer members' expectations, however, added a second layer. The initial expectations of the CME members involve setting up and facilitating meetings, including ‘to organise the content of the meetings’ (CMET04), ‘facilitate learning by people sharing their own knowledge and experience’ (CMET06) and ‘provide access to resources’ (CMET07). Over time, the initial expectations expanded, with peer members increasingly relying on the tutor for informal support and guidance.

The final layer of expectations originated from the CME tutors themselves. Although they often experienced internal pressure to be well informed and cover a broad array of topics, their primary self‐expectation was to maintain a positive, proactive attitude and foster a supportive, encouraging environment for honest and open communication:

There's always a bit of humour. There's a levity even when you could be dealing with the darkest topics. I think it's important to bring a bit of lightness into it, so that people aren't too overwhelmed or upset. (CMET05)

I think we all realised, as time went on, that everybody else was in the same boat as you. Everybody else was making the same mistakes that you were making, and everybody else was buoyed up by the fact that you were able to talk about your mistakes without being afraid of being criticised. That was the huge thing in small groups. (CMET12)

### 3.3 Navigating Role

One of the study's research questions was to identify whether role tensions were part of the work experience of CME tutors. As CME tutors have multiple roles, each with its own non‐negotiables, juggling among them to achieve balance was seen as a daily reality. One of the participants vividly described his commitment to do so:

I work seven sessions, which is three and a half days a week, and then I have a day to a day and a half of a week that I can do CME stuff when I need to, but it still leaves me time to do my GP Trainer work and teaching for my trainees and my registrars, and also be a parent or father … try and get to kids games and coaching as I am coaching kids, teams, and whatever other stuff outside of that, you're kind of busy. (CMET05)

The pressure of managing multiple responsibilities with limited time led CME tutors to experience internal tensions. The expectations and acceptance that CME meetings are scheduled after working hours leave CME tutors feeling overextended:

I travelled maybe 30 or 40 kilometres to one of the meetings, so you leave home, say goodbye to the children and my wife, and wouldn't get back until about 11 o'clock at night, or half 11. (CMET12)

I think it's just trying to juggle it all, and sometimes I just can't get it in …. (CMET02)

As family life demands presence, challenges arising from evening meetings often contribute to role strain. Although CME tutors attempted to fulfil both sets of obligations (personal and professional), occasionally some of them had feelings of guilt toward family:

It comes at a cost, because if you think about it from my point of view, it's 3 evenings a week that I'm gone from my family. It's 3 weekends a year that I'm away from my family to do the job. (CMET06)

The role strain is often navigated by developing strong organisational skills to meet the expectations and responsibilities of professional roles. The organisational skills are reflected through having strict time management, meeting deadlines and being organised:

I'm actually very good at meeting deadlines. It's one of the few talents I have. I'm very punctual, I don't like being late ever, and if there's something to be done, it has to be done by so and so. (CMET03)

Friday mornings are my time for the organisation, admin, and preparation. I try to protect that little bit of time, but there are definitely times when you do way more than that, and it balances out. (CMET04)

While reflecting further on the role, participants often identified the remuneration aspect as a potential source of strain. Most participants felt that their commitment to the role, as reflected in the time invested in preparing and running the meetings, maintaining continuous communication with CME members and supporting peer education and further development, was not adequately financially supported. Inadequate remuneration made them feel undervalued and unrecognised, as one participant echoed:

It is such an important job that people need to recognise it, for it is a senior educational post that should be respected and remunerated, and that means you'll get quality in there, people who want to do it. (CMET03)

While inadequate financial compensation contributed to significant role strain, it was navigated by strong commitment to learning, education and camaraderie with peers, ultimately enabling the positive dimension of the role to outweigh this negative aspect.

### 3.4 Evolving New Identities

While investigating reflections on professional identity, the interview findings revealed that taking on the CME tutor role guided their personal and professional development.

In particular, some tutors felt that the role helped improve their awareness of social dynamics by enhancing ‘empathy, compassion, communication’ (CMET06), ‘understanding of the different personalities’ (CMET12) and refining their ‘ability to listen’ (CMET13).

The tutor role encouraged practising regular self‐reflection and critical self‐assessment, not only as a tutor but also as a GP, as illustrated by the experiences shared by two participants:

I do think teaching for me is energising because it makes you think, it makes you look at your values, it makes you look at the way you practice, it makes you practice better, and it makes you think about it. (CMET10)

You became yourself. You became even more critical of yourself and what you were doing and what you were trying to do so that when a patient walked through the door, you were trying to do absolutely everything that you could, learning all the skills of communication, of knowledge, skills, attitude, attitudes, and all of that to apply it to each consultation. (CMET12)

Furthermore, the role also fostered a unique sense of being valued, especially reflected through the validation when peers actively seek the tutor's opinion, advice and guidance:

… after a couple of months, I start seeing the local GPs look up to you. If there's any issue going on, or any medical thing going on, they will text you, or they will give you a call. So it's a good feeling in one way …. (CMET01)

Being valued and recognised by peers and other professionals helped CME tutors feel confident and capable in their roles. This external feedback enhanced their sense of identity and increased their commitment to both clinical work and teaching. Some of the participants disclosed feeling a strong ‘sense of personal satisfaction or pride’ (CMET07), making the role meaningful to who they are.

#### 3.4.1 A ‘Go‐To Person’

Throughout the career of a CME tutor, participants echoed the development of new aspects of their professional identity—becoming a trusted ‘go‐to person’—which expands beyond the tutor role to someone who offers guidance, support and connection across a broad spectrum of issues affecting peer GPs personally and professionally. A ‘go‐to person’ identity emerged from both the tutor's own sense of responsibility to create a supportive environment and the expectations placed on them by their peers. The support often expanded beyond clinical or educational practicalities and involved offering reassurance, solidarity, and practical help in times of uncertainty:

Suddenly, other young GPs are phoning me for advice, and ‘What do you think I should do in this situation?’. It is not just clinical advice, but practice advice. So I'm suddenly being asked all sorts of questions, nothing to do with education. You know bits of advice outside of this role. (CMET08)

You end up giving all sorts of advice to people about where to live and where to get your car fixed. Something the new people in town seem to look for is every bit of knowledge about everything they can think of. (CMET09)

Undertaking a ‘go‐to person’ role provided tutors with a sense of strong leadership; however, it also imposed certain challenges. Some tutors felt that ‘the tutor role never stops’ (CMET09), as there were assumed expectations to be continually available and helpful. Although supporting peer members was found to be gratifying, it also carried a sense of responsibility that resulted in pressure and fear of not meeting others' expectations:

I have no problem with them asking me stuff, but I don't know whether I'm going to give them good advice because I worry if I've given them bad advice. (CMET08)

There is a supportive element, but it isn't a therapeutic group. The boundaries had to be clarified …. (CMET11)

## 4 Discussion

Our study explored the working experiences of CME tutors in Ireland, drawing on their reflections on their roles and professional identities. The study participants viewed their role as deeply rewarding and enriching their clinical practice, reflective skills and identity as both doctors and educators. At times, balancing multiple roles and expectations created role strain, as reflected in extended time commitments and reduced presence in family life. Embracing an informal role as a ‘go‐to person’ provided tutors with a more profound sense of value and connection with their peers; however, it also imposed additional obligations on them in areas beyond their remit.

The literature on SGL in general practice highlights the tutor or facilitator role as pivotal for creating safe, collaborative environments where GP peers can thrive and further develop necessary knowledge and practical skills [8, 19, 20, 21, 22, 23]. Motivation to take on and sustain the role stems from a sense of reward, intrinsic satisfaction, professional growth and the value placed on supporting colleagues [19, 22]. The role is often described as a shift from traditional teaching to a participatory approach, in which CME tutors share responsibility for learning and support of peer dynamics [19, 23]. The findings of our study also emphasised the benefits of the tutor role in terms of the social support and camaraderie of being part of a dynamic network of like‐minded GPs with a passion for further learning and self‐development, both personally and professionally. Previous research has also highlighted the importance of the tutor role in building social bonds and mutual trust, where peer members feel safe sharing their reflections and discussing a variety of topics [8, 22, 23]. Essentially, a well‐established tutor not only provides a space for exploration but also encourages peers to open up, connect and learn.

Over the years, the CME tutor role in Ireland has evolved, expanding beyond organisational expectations and transforming into the provision of support exceeding contractual hours. Consequently, the tutor role has become more complex and onerous than previously understood. Although GP peer support networks exist internationally and nationally [24, 25, 26], addressing psychosocial, occupational and personal challenges, our study shows that GPs continue to turn to CME tutors for support.

The additional ‘roles’ undertaken by the Irish CME Tutors are reflective of GP group members' needs, not only regarding education but also support at varying times of stress, for example, setting up in practice, being single and isolated in practice, dealing with a complaint or error. Serving as a ‘go‐to person’ or occupying a peer mentoring role can act as a significant catalyst for professional identity formation, enhanced self‐efficacy and role consolidation [27, 28, 29]. Individuals in such positions often experience reinforcement of their perceived competence, trustworthiness and leadership capacity, which contributes to strengthened self‐confidence and a more defined professional identity [27, 28, 29]. Our findings resonate with these perspectives, as participants frequently described feeling empowered by this role, highlighting not only its intrinsic appeal but also the depth of commitment and dedication consistently demonstrated by tutors. Yet, the expectation to be continually available and emotionally attuned to others can result in role strain, particularly when personal resources (e.g., time, emotional energy or expertise) are limited. Tutors may feel pressure to meet the diverse needs of peers while managing their own responsibilities, which can lead to emotional fatigue, self‐doubt or blurred boundaries between peer and authority roles. In some cases, the internalised obligation to ‘be there’ for others may hinder the tutor's help‐seeking behaviour, reinforcing a one‐directional support dynamic.

The expanded CME tutor role needs to be reflected in the job description and the support offered. To protect and support current and future CME tutors, additional guidance and policies are needed. A potential for developing a ‘buddy system’ has been noted in our study, which would enable regular and frequent check‐ups with individual CME tutors. Previous research also highlighted the need for ongoing support for CME tutors, including one‐to‐one mentorship and regular supervision meetings [19, 21]. Regular check‐ups would facilitate discussion of challenges (including workload and role strain) and ongoing reflection on teaching strategies and peer engagement. Therefore, the next steps should incorporate further revision of the CME tutor job specification and support needed to reflect the dynamic nature of the current role. An improved and more formal understanding of the CME tutor role will facilitate better induction and the development of more targeted supports for tutors.

Further review and advocacy regarding the appropriate level of remuneration is also needed. The payment received for the wide variety of tasks performed in this role, combined with evening hours and substantial additional duties, was questioned. In Ireland, the GP daytime sessional rate has improved, and other new GP roles have generated much higher remuneration packages; however, the CME tutor's sessional rate has remained unchanged. CME in Ireland is substantially funded by the HSE, supplemented by the Irish College of GPs. The sessional rate paid to tutors is at least equivalent to other non‐clinical roles; however, it would be lower than the clinical rate paid by GP practices to a GP. This pay gap between clinical and non‐clinical State‐funded rates shapes tutors' perceptions of how their work is valued within the wider system and can influence their sense of professional identity.

Improved remuneration will not only acknowledge tutors' professional expertise but also allow tutors to justify reducing their commitment to the clinical GP, unsociable hours and time away from the family, thereby aiding retention, motivation and long‐term commitment to the CME programme's success.

### 4.1 Limitations

The application of the qualitative study design helped uncover rich narratives, enabling us to delve deeper into the role of CME tutors and perceptions around their identity.

Because our study provided insights into the CME tutors' experiences at a single point in time, we believe that to gain a better understanding of the evolution of this important role, future studies would benefit from adopting a longitudinal study design. Investigating the tutor's role through changes over time (comparing reflections at the start of the career with those at retirement) or by examining progression outside the tutor role (e.g., training/clinical lead) during and after the ‘post’ period would be beneficial.

We are also aware that our sample size was relatively small. Although more CME tutors expressed interest in participating in the interview, data saturation was reached after 13 interviews, and recruitment ceased.

## 5 Conclusion

Overall, the findings in this study demonstrated that the career of CME tutors in Ireland is highly valued. The tutor role offered opportunities to develop a wide range of practical and clinical skills, as well as to provide a supportive and stimulating learning environment for their peers.

Yet, additional support is needed to address role‐related challenges and ensure the long‐term attractiveness and sustainability of this vital professional path.

## Author Contributions

**Ivana Keenan:** conceptualisation, project administration, formal analysis, methodology, investigation, writing – original draft, writing – review and editing, visualisation. **Aileen Barrett:** conceptualisation, formal analysis, methodology, writing – review and editing. **Illona Duffy:** conceptualisation, project administration, writing – review and editing.Laoise Byrne: conceptualisation, project administration, writing – review and editing.**Claire McNicholas:** conceptualisation, project administration, writing – review and editing. **Gillian Doran:** conceptualisation, project administration, writing – review and editing. **Stephanie Dowling:** conceptualisation, writing – review and editing.

## Funding

The authors have nothing to report.

## Ethics Statement

Ethics approval for the study was obtained from the Irish College of GPs Research Ethics Committee (ICGP_REC_2024_ 2803). A strong emphasis was placed on voluntary participation and the participant's right to withdraw from the research at any point without prejudice or giving reasons for that decision. Before analysis, all transcripts were de‐identified to ensure the participants' anonymity and confidentiality.

## Conflicts of Interest

The authors declare no conflicts of interest.

## Data Availability

Data are not open access to protect participant confidentiality and anonymity.

## Interview Topic Guide

1. Can you describe your journey into becoming a CME tutor?
2. Can you tell me more about your current CME tutor role?
3. What roles other than CME tutor do you currently hold?
4. How would you define your professional identity? Do you mainly see yourself as a doctor, educator or both?
5. How would you describe your identity as a tutor?
6. What do you think others expect from you as a tutor? How does this compare to what you expect from yourself?
7. Have you ever experienced difficulties balancing your role as a tutor and other professional responsibilities? How did you handle it?
8. How do you manage situations when others (e.g., the CME network, the College, Faculty, consultants, peers) have different expectations from your role?
9. Do you feel like you are part of a larger community of medical educators? How does that influence your professional identity?
10. Has your role as a tutor influenced your career path? If yes, in what way?
11. Do you see yourself continuing to work as a CME tutor in the long term?
12. What are the main challenges you experience in this role, and how do you address them?
13. What additional supports would benefit you in this role?

Is there anything else you would like to add?
